# Long segment stenosis—the narrow path to centralization in airway surgery

**DOI:** 10.1093/icvts/ivae004

**Published:** 2024-01-12

**Authors:** Clemens Aigner

**Affiliations:** Department of Thoracic Surgery, Comprehensive Center for Chest Disease, Medical University of Vienna—Vienna University Hospital, Vienna, Austria

**Keywords:** Airway surgery, Tracheal surgery, Laryngotracheal surgery, Tracheal stenosis, Resident training, Centralization

Airway surgery is typically marked by low patient numbers. Thus, high-volume centres are rare and reliable data on outcome, quality measures and training requirements are limited. The Brazilian Society of Thoracic Surgeons (BSTS) must be congratulated to conduct a survey on practice patterns in airway surgery in Brazil and thereby to provide a structured overview on caseload, perioperative management and educational aspects [1].

The median number of 5 procedures per institution with a response rate of 24% clearly demonstrates a low degree of centralization, which likely reflects the situation in the majority of countries worldwide. A granular picture of the present situation is the first step required to be able to take targeted measures and initiate change. The possibility to centralize treatment of certain disease patterns or procedure types is highly dependent on the structure of the healthcare system as well as incentives placed by the system. Centralization seems meaningful in rare complex procedures, since a relation between caseload and results is intuitive, however generally accepted outcome measures are not established and difficult to substantiate without availability of required data. A positive effect of centralization and increased caseload has already been demonstrated in more frequently performed procedures such as lung cancer surgery in several countries [2–4]. Transparency of practice patterns and outcome data are a first step. An analysis of the Society of Thoracic Surgeons General Thoracic Surgery Database identified an association of operative volume with morbidity after tracheal resection when comparing 9 institutions performing ≥4 procedures per year with 98 institutions with <4 procedures annually [5]. These results provide an argumentative basis for centralization and should be validated by further multi-institutional analyses.

Standardization of preoperative work-up, surgical technique, perioperative management and assessment of outcome are difficult to establish without sufficient case numbers. Quality control measures are therefore frequently not established. The presented survey clearly shows a lack of availability of technical equipment required to offer the highest quality of care in complex situations or for the management of complications. High-frequency jet ventilation and ECMO are valuable tools in airway surgery, however only available to 26% and 27.3% of responding institutions. Equally stents as endoscopic alternative or option for the management of complications are not readily available to the vast majority of institutions. In contrast to this, the self-declared proficiency in adult tracheal surgery, which exceeds 80% in the majority of procedures and even in laryngeal split with cartilage grafting and carinal resection approximates 60% of responding surgeons is noteworthy. Due to the overall low case numbers no reliable data on learning curves or number needed to achieve proficiency for airway surgery are available. The paper offers a perceived number of 20 procedures as the median response. In spite of the low overall numbers, 59.6% of responders consider a 2nd-year resident capable of performing an entire airway procedure under supervision. It is important to mention that the general thoracic surgery training in Brazil consists of 2 years focused on thoracic surgery after 2–3 years of training on general surgery and thus cannot directly be compared to general thoracic surgery training programs starting immediately after graduation. It is obvious that only a limited number of thoracic surgeons can be fully trained and obtain a constant exposure to airway surgery to acquaint and maintain proficiency at a high level. On the technical level, other fields in thoracic surgery such as lung transplantation clearly offer synergies with laryngotracheal surgery. A structured curriculum for residents aiming to specialize in airway surgery is warranted and several valuable items are mentioned in the discussion of the survey.

Interdisciplinary cooperation is crucial to optimize patient selection and comprehensively assess outcome. The presented results indicate that voice and swallowing function is rarely assessed beyond the assessment of respiratory limitation, even though these are crucial factors to assess functional outcome [6]. Other specialties like pulmonology and anaesthesia are equally important cooperation partners in the comprehensive management of patients with airway pathologies and the suggested implementation of an airway board should be mandatory in centres focusing on central airway surgery.

This analysis of the current Brazilian situation of airway surgery provides a valuable addition to the literature to further aim for a standardization and centralization of airway surgery with comprehensive multi-institutional outcome data assessment.
